# Evaluating the Ameliorative Potential of Quercetin against the Bleomycin-Induced Pulmonary Fibrosis in Wistar Rats

**DOI:** 10.1155/2013/921724

**Published:** 2013-12-12

**Authors:** Ramesh Verma, Lokendra Kushwah, Darpesh Gohel, Manish Patel, Tulsi Marvania, Suresh Balakrishnan

**Affiliations:** ^1^Department of Toxicology, Jai Research Foundation, Valvada, Gujarat 396108, India; ^2^Division of Toxicology, Department of Zoology, Faculty of Science, The M.S. University of Baroda, Vadodara, Gujarat 390002, India

## Abstract

The current study deals with the effect of a dietary flavanoid quercetin on fibrotic lung tissue in rats. Bleomycin was administered by single intratracheal instillation to Wistar rats to induce lung fibrosis. The pathologies associated with this included significantly reduced antioxidant capacity, ultimately leading to protracted inflammation of the lung tissue. The hallmark of this induced fibrosis condition was an excessive collagen deposition in peribronchial and perialveolar regions of the lung. Oral quercetin treatment over a period of twenty days resulted in significant reversal of the pathologies. The antioxidant defense in lung tissue was revived. Moreover, activity of the collagenase MMP-7, which was high in fibrotic tissue, was seen restored after quercetin administration. Trichome staining of lung tissue sections showed high collagen deposition in fibrotic rats, which may be a direct result of increased mobilization of collagen by MMP-7. This was appreciably reduced in quercetin treated animals. These results point towards an important protective role of quercetin against idiopathic lung fibrosis, which remains a widely prevalent yet incurable condition in the present times.

## 1. Introduction

Bleomycin is a commonly used chemotherapeutic agent which, however, induces dose-dependent pulmonary fibrosis upon long-term administration [1]. Interstitial pulmonary fibrosis is characterized by an altered cellular composition of the alveolar region with excessive deposition of collagen. However, lung inflammation is considered to be a major underlying factor for the induction of pulmonary fibrosis [2]. Reactive oxygen species such as superoxide anion, hydrogen peroxide, and hydroxyl radical are reported as major mediators of lung inflammatory processes [3]. Nevertheless, the direct linkage between reactive oxygen species formation and pulmonary fibrosis has not been established conclusively. Bleomycin-induced pulmonary injury and lung fibrosis have been documented in studies using several animal models [4, 5]. These models have been widely used for studying the mechanisms involved in the progression of human pulmonary fibrosis and the impact of various drugs on this progression [6, 7]. The bleomycin induces the genesis of reactive oxygen species upon binding to DNA and iron, which in turn causes DNA damage [8]. The interaction of bleomycin with DNA is postulated to initiate the inflammatory and fibroproliferative changes through a concerted action of various cytokines leading to collagen accumulation in the lung [5]. Further, it is reported that bleomycin promotes the depletion of endogenous antioxidant defences, thus exacerbating oxidant mediated tissue injury [8]. The lung is selectively affected by bleomycin as it lacks an enzyme that hydrolyzes the *β*-aminoalanine moiety of bleomycin, which prevents its metabolite from binding to metals such as iron [9]. Strategies aimed at reducing oxidative stress have been found to be successful in decreasing bleomycin-induced lung injury and fibrosis [10–12].

The detrimental role of reactive oxygen species in many disease states has led to the development of new antioxidants. One such group of compounds with potential antioxidant property is the flavonoids present in fruits and vegetables, of which quercetin (3,3′,4′,5,7-pentahydroxyflavone) has attracted much attention for its beneficial health effects. It has been suggested that daily intake of these substances may reduce the risk of various chronic health disorders such as cardiovascular disease, diabetes, tumor development, stroke, and neurodegenerative disease [13–16]. The beneficial effects of quercetin have been attributed to multiple mechanisms including antioxidant activity, anti-inflammation, modification of signal transduction pathways and interactions with receptors and other proteins [17]. The antioxidant activity of quercetin is primarily credited to its phenolic hydroxyl groups [18].

Beneficial health effects of quercetin against various oxidative stress related diseases have been documented [19]. However, studies examining its potential pneumoprotective effects are limited. Although experimental evidence for the role of a redox imbalance in lung fibrogenesis is substantial, this chronic disease is generally nonresponsive to conventional anti-inflammatory and immunomodulatory therapy [20]. Therefore, it was of interest to determine the ameliorative role, if any, of quercetin against bleomycin-induced lung injury because of the former's potent antioxidant activity.

## 2. Materials and Methods

### 2.1. Animals

Specific pathogen-free, healthy young adult male Wistar rats (RCCHan:WIST) of 10 to 12 weeks were used in this study. They were obtained from the Barrier Maintained Rodent Animal Breeding Facility, Jai Research Foundation, Vapi, India. All the animals were fed with Teklad certified global rat feed manufactured by Harlan, USA and UV sterilised water filtered through Kent Reverse Osmosis water filtration system was provided *ad libitum*. The rats were kept in a controlled environment (temperature: 22 ± 2°C and relative humidity: 30 to 70%) with an alternating cycle of 12 h light and dark. The animals used in this study were handled and treated in accordance with the strict guiding principles of the National Institutes of Health Guide for the Care and Use of Laboratory Animals and Association for Assessment and Accreditation of Laboratory Animal Care (AAALAC). The test facility at Jai Research Foundation is AAALAC accredited and also complies with the National Good Laboratory Practice, India. The experimental protocols were approved by the Institutional Animal Ethics Committee (approval no. 35/1999/CPCSEA).

### 2.2. Experimental Design

Rats were randomized into three groups each consisting of 10 animals [21]. Briefly, the rats were anesthetized using a combination of ketamine (80 mg/kg body weight, i.p.) and xylazine (20 mg/kg body weight, i.p.) as per standard protocol [22]. A midline incision was made in the neck and the exposed trachea was intubated with tracheal cannula under direct visualization. For induction of pulmonary fibrosis, the rats received a single dose of 6.5 U/kg body weight bleomycin sulfate dissolved in 0.5 mL of 0.9% NaCl solution on day 0 of the experiment [23]. Control rats received a single intratracheal dose of sterile saline alone. Group 1 (vehicle control) and group II (bleomycin treated) rats were treated with 0.5% carboxymethylcellulose solution orally from day 1 to day 20 of the experiment. Animals from group III were orally administered with quercetin (100 mg/kg body weight/day) in 0.5% carboxymethylcellulose solution from day 1 to day 20 of the experiment after bleomycin instillation [24]. The drug was freshly prepared in 0.5% carboxymethylcellulose solution and the concentration was adjusted so that each animal received 10 mL/kg body weight. The animals were weighed at the beginning, through and at the end of experiment. The changes in body weight were recorded.

### 2.3. Biochemical Assays

#### 2.3.1. Preparation of Lung Tissue for Biochemical Studies

On day 21 of the experiment, six animals from each group were sacrificed with thiopentone sodium and the lung lobes were excised and the tissue was used for the biochemical estimations. BAL was performed in four animals from each group under anaesthesia with thiopentone sodium and the tissue was used for the preparation of the slides to be used for histological examinations.

#### 2.3.2. Determination of Lung Hydroxyproline

The hydroxyproline assay was performed as described by Edwards and O'Brien [25]. The lung was dried and then hydrolysed at 120°C in a pressure vessel for 2–4 h. The acid hydrolysates and standards were added to 1.5 mL tubes, along with the same volume of citric/acetate buffer (citric acid, sodium acetate, sodium hydroxide, glacial acetic acid, and n-propanol pH 6.0) and chloramine T solution (chloramine T dissolved in Milli Q water). The tubes were incubated for 20 min at room temperature and Ehrlich's solution (p-dimethyl-amino-benzaldehyde, perchloric acid and n-propanol) was added to the tubes which were then incubated at 60°C for 15 min. The absorbance of the reaction product was read at 550 nm.

#### 2.3.3. Determination of Lipid Peroxidation

Malondialdehyde (MDA) is the most abundant individual aldehyde resulting from lipid peroxidation breakdown in biological systems and is commonly used as an indirect method for the estimation of lipid peroxidation. MDA content was assayed using the thiobarbituric acid test as described by Ohkawa et al. [26]. MDA reacts with thiobarbituric acid to form a coloured complex. Known amount of tissue homogenate was added to the tubes containing dodecyl sulphate, acetic acid, and thiobarbituric acid solution and heated in a water bath at 95°C for 60 minutes. Trichloroacetic acid was added to the tubes and absorbance was measured at 532 nm to determine the malondialdehyde content.

#### 2.3.4. Measurement of Superoxide Dismutase Activity

The activity of superoxide dismutase was measured following the method of Kakkar et al. [27]. A known amount of tissue homogenates was mixed with sodium pyrophosphate buffer, phenazine methosulphate, and nitroblue tetrazolium chloride. The reaction was started by the addition of NADH. The reaction mixture was incubated at 30°C for 90 seconds and stopped by the addition of glacial acetic acid. The absorbance of the chromogen formed was measured at 560 nm.

#### 2.3.5. Determination of Catalase Activity

Catalase activity was assessed by the method of Luck [28], wherein the breakdown of hydrogen peroxide is measured. Briefly, a known amount of tissue homogenate is added to 0.01% chilled digitonin and was centrifuged at 4°C and 0.05 mL of the supernatant of the tissue homogenate is mixed with 3 mL of H_2_O_2_ phosphate buffer. The change in absorbance was recorded at 30-second interval at 240 nm.

#### 2.3.6. Bronchoalveolar Lavage (BAL)

BAL fluid was obtained by the injection of 3 mL saline (three times, total 9 mL) followed by gentle aspiration of the fluid from the lungs after securing an intratracheal catheter within a trachea. With this catheter, the ratio of the recovery of lavage fluid was approximately 80% and did not significantly differ among the groups. The total numbers of cells in the bronchoalveolar lavage fluid were counted with a hemocytometer. For differential counts of leukocytes in the bronchoalveolar lavage fluid, smear slides were prepared and stained with Giemsa solution. Differential cell counts were obtained from a count of 300 cells per smear.

#### 2.3.7. Measurement of Tumor Necrosis Factor- (TNF-) *α*

TNF-*α* in plasma was assayed by specific enzyme-linked immunosorbent assay using commercially available ELISA test kits (XpressBio Life Science Products, USA). The kit contains a TNF-*α* monoclonal antibody coat for a 96-well microtitre plate and polyclonal antibody to TNF-*α*. The representative standard curve was generated using the TNF-*α* standard supplied with the kit.

#### 2.3.8. Estimation of MMP-7

MMP-7 levels in plasma and BAL were measured using an ELISA kit (R&D Systems, Minneapolis, MN, USA). The samples were added in duplicate to 96-well plates coated with the MMP-7 antibody and incubated for 2 h. After washing three times with washing buffer, the conjugated secondary antibody was added and the plate was further incubated for 2 h. Plates were washed again prior to incubation with the substrate solution for 1 h. The amplifier solution was then added, and the plate was incubated for additional 30 min. All incubation cycles were performed at room temperature. Following termination of the reaction with the stop solution; the optical density was measured at 490 nm using a microplate reader. The concentration of MMP-7 in each sample was calculated from a standard curve.

### 2.4. Histological Studies

After sacrifice, each lung tissue was perfused and fixed in 10% neutral buffer formalin and routinely processed and embedded in paraffin. Serial sections (4 *μ*m) were cut and stained with hematoxylin and eosin and Masson's trichrome for light microscopic evaluation to examine the degree of fibrosis. The severity of fibrosis was individually assessed using the semiquantitative grading system described by Szapiel [29]. According to this system, the fibrosis in lung specimens was graded as none (0) when no alveolitis was observed; mild (+)—when focal lesions occupying less than 25% of the lung was noticed in alveolar septum; moderate (++)—when widespread alveolitis involving 25–50% of the lung was observed; and severe (+++)—when a diffused alveolitis spanning more than 50% of the lung, with occasional consolidation of air spaces and patches of hemorrhagic areas within the interstitium, was observed. The entire lung section was reviewed at a magnification of s10X. Each of the 20 random microscopic fields per section was detected; a score ranging from 0 to 3 was assigned. All assessments were performed in double-blind fashion.

### 2.5. Material

Quercetin, Chloramine-T and Hydroxyproline were procured from Sigma-Aldrich Chemie GmbH. Bleomycin hydrochloride was procured from the local market and was in the form of bleomycin ampoules (15 units) manufactured by Biochem Pharmaceutical Industries Ltd., Mumbai, India. All other chemicals were of analytical grade and procured from reputed manufactures of India, namely, Sisco Research Laboratories Pvt. Ltd., Qualigens Fine Chemicals Pvt. Ltd., and HiMedia Laboratories Pvt. Ltd.

### 2.6. Statistical Analysis

Statistical analyses were carried out by analysis of variance (ANOVA) followed by Dunn's post hoc test. All analyses of data were performed using SPSS for windows version 12.0 and a probability value of 0.05 or less was considered as statistically significant.

## 3. Results

### 3.1. Changes in Body Weight

**Figure 1:**
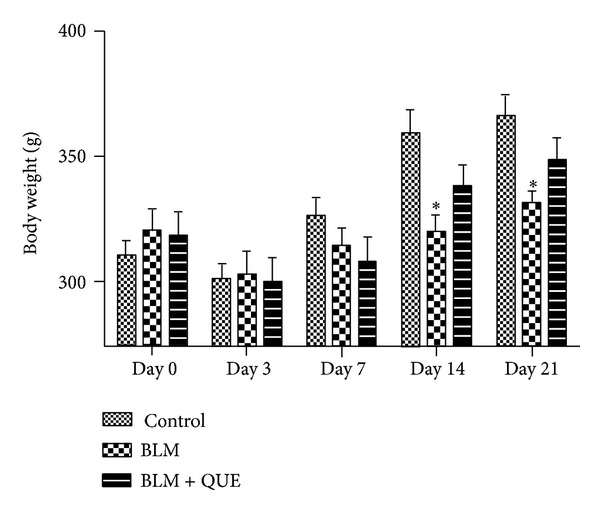
Effect of quercetin (QUE) on body weight of bleomycin- (BLM-) treated rats. *n* = 10.  *P < 0.05 versus control group.

Figure 1 shows the effect of quercetin on the body weight of bleomycin administered groups of rats. Single intratracheal administration of bleomycin (6.5 U/kg) resulted in a marked decrease in their body weight on days 14 and 21 as compared to the saline-treated control group possibly because of severe tissue damage caused by free radicals. However, body weight of quercetin-treated rats remained comparable to the control group rats throughout the period of experiment.

### 3.2. Change in Percent Relative Lung Weight

**Figure 2:**
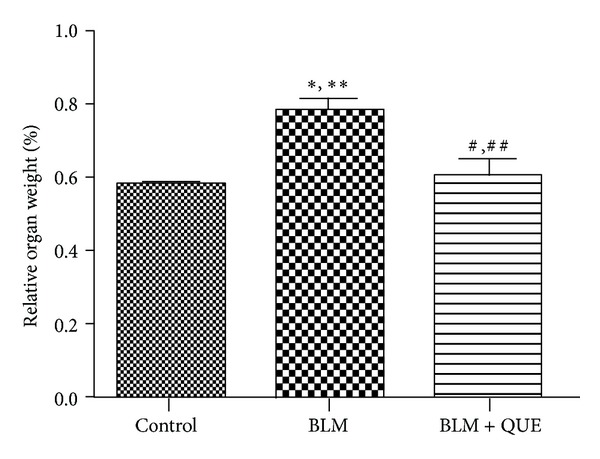
Relative weight of rat lung on day 21 of treatment. *n* = 6. *P ≤ 0.05, **P ≤ 0.01 versus control group. ^#^ *P* ≤ 0.05, ^##^ *P* ≤ 0.01 versus bleomycin group.

As evident from Figure 2, the percent relative lung weight of bleomycin-treated animals remained significantly high compared to the other two groups of animals. However, twenty-day quercetin treatment protected the lung tissue from bleomycin-induced fibrotic response as evident from the statistically comparable mean percent relative lung weight in both quercetin-treated as well as control animals.

### 3.3. Hydroxyproline Content

**Figure 3:**
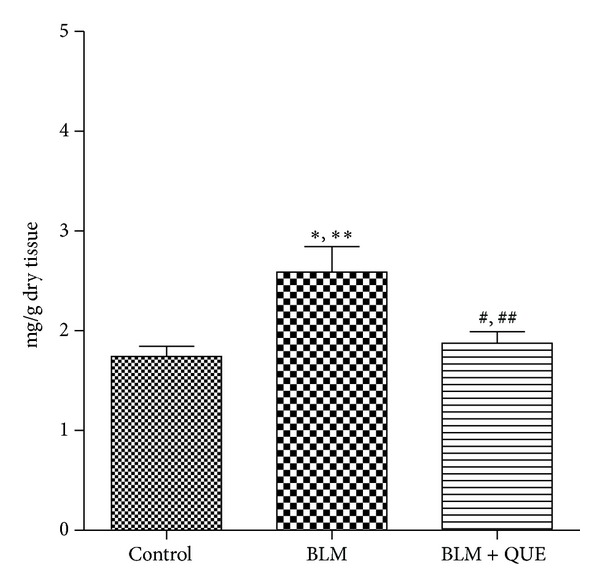
Hydroxyproline content in lung tissue on day 21 of treatment. *n* = 6.  *P ≤ 0.05, **P ≤ 0.01 versus control group. ^#^ *P* ≤ 0.05, ^##^ *P* ≤ 0.01 versus bleomycin group.

The effect of quercetin on the hydroxyproline content of lung homogenate is presented in Figure 3. Measurement of hydroxyproline is an efficient index of collagen deposition since collagen contains significant amount of this amino acid. After 20 days, the hydroxyproline content in the lungs of bleomycin group increased significantly compared to that in the control group. However, an apparent reduction (*P* ≤ 0.05) in lung hydroxyproline content was observed in the bleomycin plus quercetin group compared to the bleomycin group, indicating the possible protective role of quercetin against lung collagen deposition.

### 3.4. Lipid Peroxidation

The result of this study showed an increase in the level of lipid peroxidation in bleomycin-administered group over that of control group, which could be a manifestation of bleomycin induced tissue injury and damage. Nevertheless, quercetin treatment significantly lowered the bleomycin-induced lipid peroxidation in the lung of rats as evident from the near normal MDA levels (Table 1).

### 3.5. Superoxide Dismutase Activity

Effect of bleomycin and bleomycin plus quercetin on lung tissue superoxide dismutase activity is presented in Table 1. The superoxide dismutase activity in the lung of bleomycin-treated rat was found significantly reduced as compared to the control group. Administration of quercetin proved to be effective in restoring the altered activity of this antioxidant enzyme.

### 3.6. Catalase Activity

The catalase activity in the lung homogenate of bleomycin-treated rats was found considerably lower than that of vehicle control rats. The quercetin administration which improved the catalase activity on day 21 of treatment of the animals in this group showed comparable catalase activity with that of controls (Table 1).

### 3.7. Total and Differential Leukocyte Count in BAL

The total leukocyte count and the subset proportion in the bronchoalveolar lavage of control and experimental group of rats are given in Table 2. Bleomycin induction caused a statistically significant (*P* ≤ 0.05) increase in the total leukocyte count in the BAL as compared to that in control animals. However, in rats treated with quercetin, total leukocyte count remained similar to that observed in control rats at the end of experimental regime. The differential cell count showed a significant increase in proportion of neutrophils and eosinophils in the lungs of rats exposed to bleomycin. It was observed that the quercetin treatment for 20 days significantly reduced the bleomycin induced hike in neutrophils in the BAL. While the percentage of lymphocytes and alveolar macrophages was decreased in bleomycin-induced group, treatment with quercetin reversed these changes significantly.

### 3.8. Tumor Necrosis Factor- (TNF-) *α* Concentration

**Figure 4:**
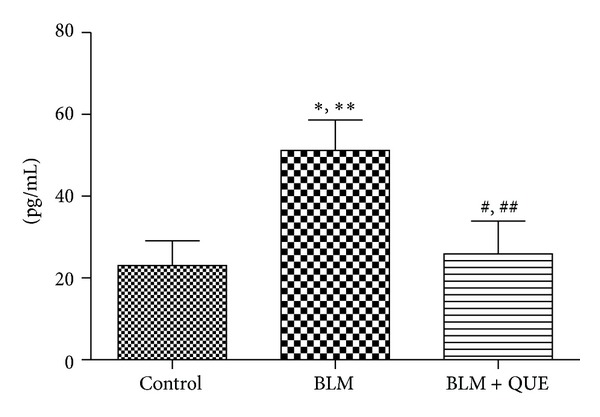
Plasma levels of tumor necrosis factor-*α* on day 21 of treatment. *n* = 10.*P ≤ 0.05, **P ≤ 0.01 versus control group. ^#^ *P* ≤ 0.05, ^##^ *P* ≤ 0.01 versus bleomycin group.

Figure 4 shows the effect of quercetin on plasma levels of TNF-*α*. The plasma TNF-*α* level of bleomycin-administered rats remained elevated on day 21 of experiment when compared with the sham-treated group. Treatment with quercetin, however, decreased bleomycin-induced increase in plasma TNF-*α* level by the end of the experiment.

### 3.9. Estimation of MMP-7

The levels of MMP-7 were estimated in blood plasma and bronchoalveolar lavage fluid in the various stated experimental groups with the help of an ELISA-based method. Our results (Table 3) showed a significant increase in the levels of MMP-7 in both plasma and bronchoalveolar lavage fluid in response to bleomycin challenge. The level of this extracellular matrix digesting enzyme in animals that received quercetin treatment after bleomycin administration was found to be markedly lower than that in the bleomycin-treated group, and, at the same time, comparable to that in the control group.

### 3.10. Histopathological Examination of Lung Tissue

Histopathological abnormalities in lungs were studied at the end of the experiment using hematoxylin and eosin staining as well as Masson's trichrome staining.

**Figure 5:**
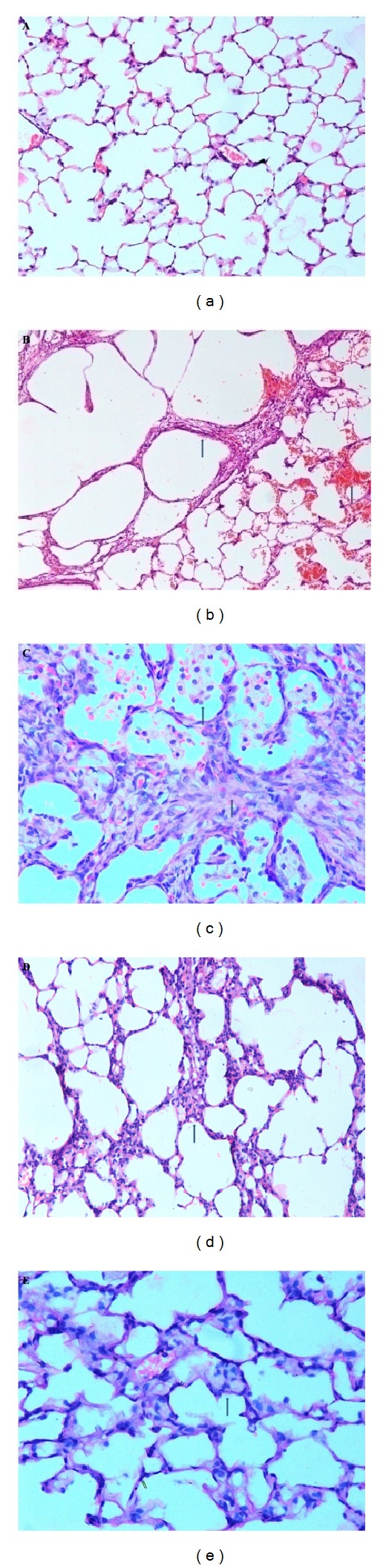
(Hematoxylin and eosin staining.) (a) Normal histoarchitecture of lung 200x. (b) Bleomycin-treated lung showing thickening of alveolar septa with hemorrhages and marked exphysematous change 200x. (c) Bleomycin-treated lung showing severe thickening of alveolar septa with fibroblast proliferation and infiltration of foamy macrophages and mononuclear cells 400x. (d) Quercetin-treated lungs showing mild to moderate thickening of alveolar septa 200x. (e) Quercetin-treated lungs showing mild thickening of alveolar septa with few inflammatory cells 400x.

Bleomycin administered rats showed distorted architecture of the lung tissue which included moderate to severe hemorrhages, emphysema, areas of increased thickening of alveolar septa, leukocytic infiltration in alveolar walls, and fibroplasia (Figures 5(b) and 5(c)) when compared with control group (Figure 5(a)). Nevertheless, querctin showed to have ameliorative effect on the inflammatory lesions developed by bleomycin treatment. Pulmonary histoarchitectural changes in animals treated with quercetin showed mild to moderate degree of septal thickening with few inflammatory cells. Emphysematous changes and alveolar hemorrhages were remarkably reduced in querctin-treated group of animals (Figures 5(d) and 5(e)).

**Figure 6:**
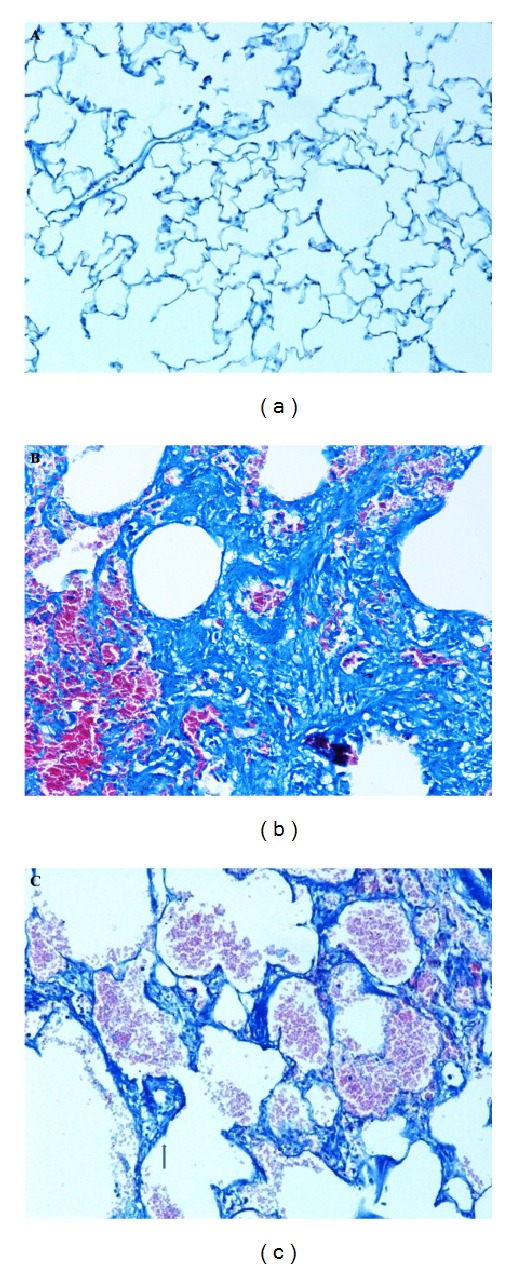
(Masson's trichrome staining.) (a) Normal histoarchitecture of lung 200x. (b) Bleomycin-treated lung showing excessive collagen deposition leading to septal thickening and compressed alveoli 200x. (c) Quercetin-treated lungs showing mild to moderate collagen deposition 200x.

In order to understand the degree of lung fibrosis in various treatment groups, the extent of collagen deposition was studied as a marker of fibrosis through Masson's trichrome staining. As expected, bleomycin-treated group displayed an increased grade of collagen deposition, compressed alveoli and large fibrotic areas, compared to that in the control group (Figures 6(a) and 6(b)). However, collagen accumulation was remarkably decreased in rats from quercetin-treated group (Figure 6(c)) when compared to the bleomycin group.

Furthermore, the semiquantitative assessment of fibrosis in lung sections was performed by scoring pathological lesions as per the Szapiel method of examination (Table 4). The Szapiel score of bleomycin-induced group was found significantly higher on day 21 when compared with control group. However, Szapiel scores on day 21 of quercetin treated group showed marked decrease compared to the bleomycin-treated group reaffirming protective role of quercetin against bleomycin-induced lung fibrosis.

## 4. Discussion

The clinical use of bleomycin, as an anticancer drug for a myriad of human malignancies, has been hampered due to its detrimental effects [30]. The major side effect is the induction of lung fibrosis in patients treated with bleomycin. Pulmonary fibrosis is commonly progressive and essentially an untreatable disease with an increasingly fatal outcome [31]. In order to understand the finer mechanisms of development of pulmonary fibrosis as well as to screen the efficacy of various compounds as potential therapeutic agent against this pathological manifestation, animal experimentations are inevitable. The bleomycin-rodent animal model of lung fibrosis is an established and widely used surrogate model of human lung fibrosis [32]. Comparison studies of patients with lung pneumopathy and experimentally induced lung fibrosis animal model have validated effectiveness of this experimental system as an acceptable model [33]. Subsequently, the use of this animal model has helped in partly establishing the pathways of lung damage leading to fibrosis [33].

In the current study, we have used Wistar rat model of lung fibrosis created by challenging the rats with a single dose of bleomycin sulfate by intratracheal instillation. A marked reduction in the body weight was observed in the bleomycin-treated group, which could be attributed to the progression of the fibrosis [34]. Twenty-one days after single intratracheal installation of bleomycin, an obvious increase in the relative weight of lungs was observed in the experimental animals compared to that of control rats. Soumyakrishnan and Sudhandiran [12] have also reported a similar increase in lung weight in bleomycin-treated animals. This increase in lung weight is a clear indication of lung fibrosis that is characterised by excessive deposition of collagen as evident from the increased hydroxyproline levels observed in the lungs of bleomycin-treated rats. However, it was noticed that administration of quercetin effectively thwarted the progression of bleomycin-induced lung fibrosis, and, hence, body weight and relative weight of the lungs in the animals of quercetin-treated group was in concurrence with that of vehicle control and remained within 95% confidence limits.

Further, the local tissue response to bleomycin-induced lung injury was evaluated by assessing various biochemical markers, namely, hydroxyproline—an index of collagen deposition, malondialdehyde—as a measure of lipid peroxidation and superoxide dismutase, as well as catalase—two key antioxidant enzymes in the lungs of rats from various study groups. In order to complement the biochemical response of bleomycin-challenged lung tissue, cytological analyses such as total and differential leukocyte counts in bronchoalveolar lavage fluid were also conducted. In addition, histochemical localization of collagen in lung tissue was done to confirm the extent of lung fibrosis in various groups of animals. Lung histopathology also was done to confirm the progression of lung fibrosis in bleomycin-instilled rats and also to unravel the possible inhibitory potential of quercetin against the bleomycin-induced lung fibrosis.

Since the amino acid hydroxyproline is the precursor for collagen, the estimation of this amino acid following acid digestion of collagen is considered a good biochemical index of collagen content. Bleomycin-treated group exhibited statistically significant increase in lung hydroxyproline content as compared to control group. This result is in accordance with previous reports, which also demonstrated remarkable increase in lung hydroxyproline content in bleomycin-instilled pulmonary fibrosis models [2, 35, 36]. Deposition of excess or abnormal collagen is a characteristic of lung fibrosis as stated by many while studying the mechanisms behind xenobiotic-induced lung fibrosis [37–39]. The present biochemical indication of bleomycin-induced lung fibrosis was further confirmed visually by collagen-specific Masson's trichrome staining of lung sections. The microscopical observation of the trichrome stained histological sections revealed that bleomycin treatment induced collagen accumulation and deposition in peribronchial and perialveolar tissues that obliterated alveolar spaces as tiny fibrils. However, the lung tissues from quercetin-treated rats showed much improved histological profile with near normal collagen deposition and a basal level of hydroxyproline content indicating quercetin's ameliorative role against bleomycin-induced pulmonary fibrosis.

Further, it is known that reactive oxygen species play an important role in the development of fibrotic responses in the lung of the subject upon bleomycin challenge. Bleomycin binds to iron (FeII), undergoes redox cycling, and catalyzes the formation of reactive oxygen species (11). It is well documented that these free radicals, once produced, target biomacromolecules such as DNA, protein, and lipid, with the ultimate progression of lipid peroxidation, resulting in damage to the lung [40]. In the current study too, we observed signs of oxidative stress as exemplified by heightened MDA activity in the lungs of animals subjected to bleomycin. Moreover, the drug-treated rats also showed compromised antioxidant response as evident from subdued activity of antioxidant enzymes such as catalase and superoxide dismutase. Similar observations made by others give credence to the present notion [41, 42]. However, it was observed that the extent of oxidative damage was well within the normal level in the lungs of quercetin-treated rats signifying this flavonoid's antioxidant potential. Flavonoids are reported to be powerful antioxidants providing remarkable protection against oxidative stress and free radical damage [13]. Moreover, quercetin, a member of the flavonoid family, is cited as one of the most prominent dietary antioxidants [13]. A study regarding the tissue distribution of quercetin in rats has shown that, upon quercetin treatment, the highest accumulation of this flavonoid and its metabolites was observed in rat lungs [43]. Hence, it is prudent to presume that quercetin could emerge as a pneumoprotective agent against local oxidative stress inducers like the one that is addressed in the current study.

In addition to the oxidative stress mentioned earlier, intratracheal administration of bleomycin might also results in interstitial inflammation characterised by an increase in the recruitment of leukocytes [44]. It is known that the recruitment of inflammatory cells to the site of inflammation plays a pivotal role in the pathogenesis of several inflammatory conditions. Further, it is also reported that the leukocytes such as macrophages, neutrophils, and lymphocytes play a key role in inflammation and tissue remodelling [44]. In the current study, a significant increase in the total leukocytes count with a marked increase in the proportion of neutrophils and eosinophils was apparent in the bronchoalveolar lavage of bleomycin treated animals. At the same time, the proportion of macrophages and lymphocytes was found low in the bronchoalveolar lavage fluid of bleomycin-challenged rats. The observed trend in the count of inflammatory cells in the bronchoalveolar lavage fluid during bleomycin induced pulmonary injury is in accordance with the previous reports of experimental pulmonary fibrosis [11, 45]. However, the anti-inflammatory property of quercetin gained further consolidation from our observation that twenty-day repeated treatment of this flavonoid after intratracheal instillation of bleomycin significantly reduced the recruitment of leukocytes into the alveolar air space. Analysis of extravasated cells from lung revealed that the recruitment of neutrophils was significantly reduced in the quercetin-treated rats compared to bleomycin-administered ones. The obvious reduction in the extravasation of leukocytes upon quercetin treatment indicates substantial recovery from bleomycin-induced lung inflammation, and hence, underlining the ameliorative property of this flavonoid against the drug-induced lung injury.

Moreover, it is reported that TNF-*α*, a potent proinflammatory cytokine, acts as a major component of a multifaceted network of cellular and molecular interactions that regulate the progression of fibrotic process [46]. In this study, a significant elevation in the expression of TNF-*α* was observed in the bleomycin-treated group, which is in accordance with previous findings [2, 47]. Furthermore, it has been reported that the reactive oxygen species generated as a result of bleomycin treatment might induce nuclear factor *κ*-B and increase the synthesis of TNF-*α* [48, 49]. However, we noticed that oral administration of quercetin for twenty-days after local instillation of bleomycin reduced the expression of proinflammatory cytokine TNF-*α* to the basal level indicating marked recovery from the induced lung injury caused by the drug in question.

Further, it is well documented that fibrosis is a consequence of repair of damaged tissue and is known to be closely associated with the remodelling of extracellular matrix tissue [50]. Also, as has been reported by others, pulmonary fibrosis patients show elevated levels of MMP-7 [51–53]. Moreover, it has been reported that mice lacking MMP-7 are protected from bleomycin-induced lung injury suggesting a key role of MMP-7 in the induction of pulmonary fibrosis by bleomycin [53]. On the line of these reports, we took up the estimation of MMP-7 to throw light on a possible role of this enzyme as a direct or indirect target of quercetin. Our results indeed do suggest an association of MMP-7 levels with induction of fibrosis by bleomycin. This increase in expression was found to be prevented by quercetin treatment. Whether MMP-7 is a direct target of quercetin, however, needs to be assessed.

The subsequent corroboratory histopathological observation showed abnormal histological profile of the lung tissue in bleomycin-administered rats. Notable deviant histoarchitecture of the lung from the drug-treated animals includes collapsed alveolae, thickened alveolar wall, and abnormal collagen deposition. Similar structural changes reported by others from bleomycin-induced lung fibrosis rat models consolidate the credence of the present experimental model [22, 40, 54]. Moreover, in the present study, it was observed that quercetin treatment decelerated the progression of bleomycin-induced structural deformation as exemplified by the low Szapiel scores in this group of animals compared to bleomycin-treated ones. It is well documented that quercetin is a strong free radical scavenger and also a good metal chelator [55]. It has also been reported that quercetin acts through various mechanisms including the antioxidative activity, the inhibition of enzymes that activate carcinogens, the modification of signal transduction pathways, and interactions with receptors and other proteins [17]. *In vitro* studies have demonstrated that quercetin, in fact, inhibited the production of reactive oxygen species in lipopolysaccharide-stimulated Kupffer cells [56]. Moreover, reports have also shown that quercetin treatment effectively reduced superoxide anions [57] and could inhibit lipid peroxidation, and hence, alterations in lung morphology during pulmonary injury or infection [57, 58].

MAPK pathways such as ERK1/2 and JNK 6 are activated in response to a high oxidative status, leading to increase in the levels of MMP-7 [59]. The MAPK pathway is a known target for potential fibrosis therapy as several fibrogenic cytokines signal through MEK/ERK, including noncanonical TGF-*β*, PDGF, IL-13, and TNF-*α* [60]. We, therefore, suggest that quercetin, due to its antioxidative property and inhibitory effect on TNF-*α*, targets the above mentioned pathway to ultimately decrease the MMP-7 levels in quercetin-treated rats.

In the light of the current observations as well as the above cited reports, it is pertinent to presume that quercetin, being an antioxidant, effectively reduces the formation of bleomycin-induced free radicals which are known to trigger a cascade of inflammatory responses culminating in moderate to severe lung injury in treated subjects, and hence, could be used as an effective inhibitor of the bleomycin-induced pulmonary fibrosis.

## 5. Conclusion 

Pulmonary fibrosis is generally nonresponsive to conventional therapy. The bleomycin-induced fibrosis model displayed reduced antioxidant capacity, elevation of inflammatory cytokines, increased MMP-7 expression, fibrotic changes, and collagen accumulation in lung tissue. Quercetin appeared to have a pneumoprotective effect through enhancement of antioxidant status, decrease in the level of inflammatory cytokines, reduction of MMP-7 expression, and minimisation of collagen accumulation. We, therefore, suggest that quercetin effectively attenuates the pulmonary injury induced by bleomycin challenge. Additional studies, however, will be required to understand the comprehensive role of quercetin in the pathogenesis of pulmonary fibrosis.

## Figures and Tables

**Table 1:** Levels or activities of various markers of oxidative stress in the lung tissue of the rats from control and treated groups.

Biochemical estimations	Control	Bleomycin	Bleomycin + quercetin
Malondialdehyde level (nmoles MDA/mg tissue)	44.34 ± 1.28	60.63 ± 1.87*	46.64 ± 1.13^#^
Superoxide dismutase (U/mg tissue)	0.34 ± 0.03	0.21 ± 0.01*	0.31 ± 0.02^#^
Catalase (*μ*M H_2_O_2_ consumed/mg tissue/min)	18.94 ± 1.22	12.68 ± 0.82*	17.32 ± 1.46^#^

Values are given as mean ± SE for groups of six rats each, *P ≤ 0.05 versus control group, ^#^ *P* ≤ 0.05 versus bleomycin group.

**Table 2:** Total leukocyte count and percentage leukocyte subset in the bronchoalveolar lavage fluid of rats from control and treatment groups.

Experimental group	Total cells (×10^6^ mL^−1^)	Macrophage (%)	Neutrophils (%)	Eosinophil (%)	Lymphocytes (%)
Control	0.65 ± 0.12	85.17 ± 2.00	1.83 ± 0.50	0.83 ± 0.17	11.67 ± 1.50
Bleomycin	1.64 ± 0.15*	49.33 ± 3.83*	38.33 ± 1.83*	4.08 ± 0.58*	5.42 ± 0.92*
Bleomycin + quercetin	0.87 ± 0.14^#^	71.00 ± 3.00^∗,#^	13.00 ± 2.00^∗,#^	4.08 ± 0.96*	10.50 ± 1.25

Values are given as mean ± SE for groups of four rats each, *P ≤ 0.05 versus control group, ^#^ *P* ≤ 0.05 versus bleomycin group.

**Table 3:** Level of MMP-7 in blood plasma and bronchoalveolar lavage fluid.

Treatment groups	MMP-7 (ng/mL)	
	BAL	Plasma
Control	7.65 ± 0.15	5.59 ± 0.19
Bleomycin	10.58 ± 0.56*	7.28 ± 0.49*
Bleomycin + quercetin	8.16 ± 0.25^#^	6.19 ± 0.17^#^

Values are given as mean ± SE for groups of four rats each, *P ≤ 0.05 versus control group, ^#^ *P* ≤ 0.05 versus bleomycin group.

**Table 4:** Szapiel examination scores of lung tissue from rats subjected to various treatments.

Experimental group	Grade of fibrosis
Control	0.00 ± 0.00
Bleomycin	2.51 ± 0.06*
Bleomycin + quercetin	1.48 ± 0.15^∗,#^

Values are given as mean ± SE for groups of six rats each, *P ≤ 0.05 versus control group, ^#^ *P* ≤ 0.05 versus bleomycin group.
